# Divergent Impact of Glucose Availability on Human Virus-Specific and Generically Activated CD8 T Cells

**DOI:** 10.3390/metabo10110461

**Published:** 2020-11-13

**Authors:** Jenifer Sanchez, Ian Jackson, Katie R. Flaherty, Tamara Muliaditan, Anna Schurich

**Affiliations:** 1 Department of Infectious Diseases, School of Immunology and Microbial Sciences, Guy’s Hospital, King’s College London, London SE1 9RT, UK; jenifer.sanchez@kcl.ac.uk (J.S.); katie.flaherty@kcl.ac.uk (K.R.F.); 2 Peter Gorer Department of Immunobiology, School of Immunology and Microbial Sciences, Guy’s Hospital, King’s College London, London SE1 9RT, UK; ian.jackson@kcl.ac.uk; 3 Leucid Bio Ltd., Guy’s Hospital, Great Maze Pond, London SE1 9RT, UK; t.muliaditan-dekoning@uu.nl; 4 Department of Pharmaceutical Sciences, Faculty of Science, Utrecht University, David de Wiedgebouw, Universiteitsweg 99, 3584 CG Utrecht, The Netherlands

**Keywords:** glucose metabolism, virus-specific CD8 T cell, culture condition, EBV, influenza

## Abstract

Upon activation T cells engage glucose metabolism to fuel the costly effector functions needed for a robust immune response. Consequently, the availability of glucose can impact on T cell function. The glucose concentrations used in conventional culture media and common metabolic assays are often artificially high, representing hyperglycaemic levels rarely present in vivo. We show here that reducing glucose concentration to physiological levels in culture differentially impacted on virus-specific compared to generically activated human CD8 T cell responses. In virus-specific T cells, limiting glucose availability significantly reduced the frequency of effector-cytokine producing T cells, but promoted the upregulation of CD69 and CD103 associated with an increased capacity for tissue retention. In contrast the functionality of generically activated T cells was largely unaffected and these showed reduced differentiation towards a residency phenotype. Furthermore, T cells being cultured at physiological glucose concentrations were more susceptible to viral infection. This setting resulted in significantly improved lentiviral transduction rates of primary cells. Our data suggest that CD8 T cells are exquisitely adapted to their niche and provide a reminder of the need to better mimic physiological conditions to study the complex nature of the human CD8 T cell immune response.

## 1. Introduction

Glucose is a key metabolite for rapidly dividing cells. Upon T cell receptor-mediated activation, T cells undergo metabolic reprogramming, switching from predominantly relying on oxidative phosphorylation (OXPHOS) fuelled by fatty acid oxidation, to significantly upregulating glycolysis to sustain metabolically demanding effector functions and proliferation [1]. Despite the net ATP production from glycolysis being low compared to OXPHOS, glycolysis yields an important carbon source for the generation of nucleic acids, amino acids and phospholipids while leaving biosynthetic building blocks, such as fatty acids and amino acids, intact for being incorporated into cellular components [2]. Furthermore, glycolysis has a direct impact on the production of the key T cell effector cytokine, interferon gamma (IFN-γ), by promoting expression through epigenetic changes [3].

It is well recognized that T cell responses are vital for successful protection against viral infection. While resolution of an acute infection is usually followed by the formation of long-lived memory T cells, persistent stimulation of virus-specific T cells in chronic infection leads to altered T cell function and can result in the development of T cell exhaustion [4]. T cell exhaustion, which is also a main driver of failed T cell responses in cancer, is characterized by a sequential loss of function and increased expression of inhibitory receptors such as programmed death 1 (PD-1) [4]. We and others have demonstrated that the state of T cell exhaustion is accompanied by alterations in glucose metabolism and defects in mitochondrial function [5,6,7,8,9]. In tissues, long-lasting antiviral immunity is conferred by resident memory T cells (T_RM_). These are identified by expression of CD69 and CD103, molecules that promote the retention of T cells within tissues [10,11,12]. In a number of organs including the liver, T_RM_ express high levels of PD-1 despite remaining highly functional and metabolically active. Importantly CD8 T_RM_ contribute to the control of chronic hepatitis B virus (HBV) infection [10,13] and can phenotypically and metabolically adapt to their local microenvironments supporting their survival.

At sites of inflammation, the influx of activated immune cells and the metabolic activity of the recruited cells, especially neutrophils, can lead to a local decrease in glucose levels [14]. Furthermore, viruses have been reported to promote metabolic changes in their host cells, for example driving increased glycolysis or glutamine metabolism, which is advantageous for viral propagation. In addition, this also has the potential to further deplete the host cell’s microenvironment of essential nutrients. Thus, viruses can create their own niche, allowing viral persistence and propagation while the immune response is attenuated [4,15,16]. A similar mechanism is found in the tumour microenvironment in which metabolically active tumour cells deplete nutrients and can significantly lower glucose availability, thereby inhibiting CD4+ Th1 and CD8+ effector T cell function [17]. Experimentally, pharmacological inhibition of glycolysis by the glucose analogue 2-deoxy-d-glucose (2DG) dramatically affects T cell effector functions, impairs TCR signalling and alters T cell proliferation [18].

In contrast, glucose concentrations used in conventional culture media and common metabolic assays are often maintained at 10 mM, representing hyperglycaemic levels rarely present in vivo. The physiological glucose concentrations in the body are tightly controlled and range in the blood between 4.5 and 5.2 mM at the fasting stage in humans [19], but can vary locally within the tissue environment depending on cellular glucose consumption [20]. Perturbations in systemic glucose levels are often associated with disease, with levels between 6 and 9 mM being considered prediabetic and symptoms of hyperglycaemia becoming apparent at levels above 10 mM [21]. On the other hand, glucose concentrations below 3 mM are clinically considered hypoglycaemic. In this present study we tested the hypothesis that T cell responses would be influenced by alterations to glucose concentration within the physiological range.

Using two of our standard CD8 T cell assays, we investigated: (i) generic activation with plate bound anti-CD3 antibody and (ii) virus-specific responses using a selection of immunodominant virus-derived HLA class 1 restricted peptides. As target viruses we chose two ubiquitous human infections to study in detail: Epstein–Barr virus (EBV), estimated to chronically infect 90% of the world-wide population [22] and influenza A, as an example of an acute resolved infection, which most adults will have been exposed to.

We present data demonstrating that lowering the glucose concentration to a physiological level had a profound effect on virus-specific T cell responses, reducing the number of effector cytokine producing cells and promoting a resident memory phenotype. Virus-specific CD8 T cell responses deviated significantly from those of generically activated T cells, with those specific to EBV being the most distinct. Finally, T cells stimulated at physiological glucose concentrations were more susceptible to transduction by lentiviral vectors a feature that could be exploited in the experimental settings but could also shed light on mechanisms of T cell-targeting viral infections. 

## 2. Results

### 2.1. Polyclonal T Cells Have a Distinct Activation Profile to Virus-Specific CD8 T Cells upon T Cell Receptor Mediated Stimulation

**Figure 1:**
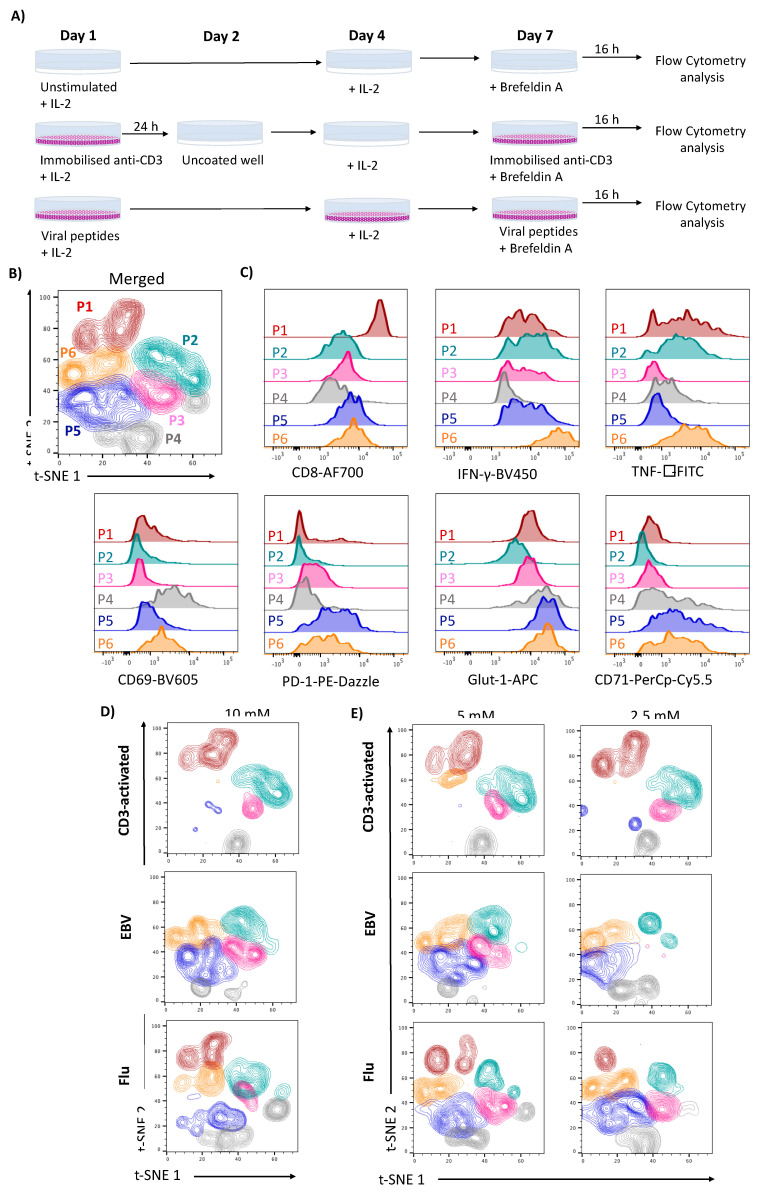
CD3-activated and virus-specific T cells have a different activation profile. PBMCs were either stimulated overnight with plate-bound anti-CD3, then rested in non-coated wells and restimulated overnight with 1 µg/mL coated anti-CD3 on day 7 or were stimulated with 0.5 µg/mL of Epstein–Barr virus (EBV) or flu peptides, cultured for 7 days and restimulated with viral peptides overnight on day 7. All T cells were maintained in media containing IL-2, which was replenished on day 4. Cytokine production and expression of specific surface markers were investigated by flow cytometry on day 8 post restimulation. (**A**) Diagram of the experimental protocol followed. (**B**) t-SNE algorithm was used to distribute responding cells defined as IFN-γ^+^CD8^+^ T cells from the anti-CD3, flu and EBV stimulated cells from the same donor in different populations depending on the expression of specific surface/intracellular markers. (**C**) Histograms that show the expression of specific surface markers and cytokines used to define the following 6 populations of IFN-γ^+^CD8^+^ T after overnight restimulation: population 1 (P1) CD8^high^ IFN-γ^high^TNF-α^high^ CD69^low/neg^ PD-1^low/neg^ Glut1^high^ CD71^neg^, population 2 (P2) CD8^int^ IFN-γ^high^ TNF-α^high^CD69^neg^ PD-1^neg^ Glut1^int^ CD71^neg^, population 3 (P3) CD8^int^ IFN-γ^int^ TNF-α^neg^ CD69^neg^ PD-1^int^ Glut1^int^ CD71^neg^, population 4 (P4) CD8^low^ IFN-γ^low^ TNF-α^low^ CD69^high^ PD-1^low^ Glut1^high^ CD71^int^, population 5 (P5) CD8^int^ IFN-γ^high^ TNF-α^high^ CD69^low/neg^ PD-1^high^ Glut1^high^ CD71^high^ and population 6 (P6) CD8^int^ IFN-γ^high^ TNF-α^high^ CD69^int^ PD-1^int^Glut1^high^ CD71^high^ (**D**) Clusters of populations detected using t-SNE algorithm after different stimulations in media containing 10 mM and (**E**) 5 and 2.5 mM of glucose.

We first activated T cells in conventional RPMI 1640 medium (glucose concentration 11.1 mM) using either generic activation with immobilized anti-CD3 (in the following referred to as CD3-activated) or using a selection of immunodominant peptides derived from two common human viral infections; Epstein–Barr Virus (EBV) as an example of a chronic infection and Influenza A virus (Flu) as an example of an acute resolved infection. To expand and study virus-specific T cells in culture we used a well-established stimulation protocol [23] as schematically outlined in Figure 1A. T cells are heavily reliant on IL-2 for function and differentiation [24] and therefore a low level of 20 IU/mL IL-2 was maintained throughout (schematic Figure 1A). Responding virus-specific T cells and CD3-activated T cells were defined by production of the effector cytokine and antiviral mediator IFN-γ, detected upon restimulation by intracellular cytokine staining as described previously [6]. We then used the t-distributed stochastic neighbour embedding (t-SNE) algorithm [25] to compare the phenotype of the IFN-γ positive CD8 populations in response to either viral peptide or upon polyclonal stimulation. Assessing the production of IFN-γ and TNF-α along with the extent of CD8 expression, the activation marker CD69, the activation and coinhibitory marker programmed death-1 (PD-1), the nutrient transporter glucose transporter-1 (Glut1) and the iron transporter transferrin receptor 1 (TfR1 and CD71), we noted that the cells fell into six identifiable populations (P1-P6; Figure 1B,C). CD3-activated, EBV and Flu specific CD8 T cells showed distinct segregation patterns (Figure 1D). Notably, the subpopulation denoted as P1, characterized by high expression level of CD8, was absent in IFN- producing EBV-specific T cells, while P5 and P6, both characterized by increased PD-1, Glut1 and CD71 expression, appeared altered in CD3-activated T cells (Figure 1D), flu-specific T cells were the most diverse containing all six populations, suggesting that different cell populations were reactivated by the three stimuli tested.

Production of the antiviral T cell effector cytokine IFN-γ is directly supported by glycolytic activity [3,26]. Pharmacological inhibition of glycolysis or glucose restriction impairs the production of this cytokine [27], whilst moderately high glucose levels around 8 mM promote IFN-γ production [28]. We reasoned that the high glucose concentrations in standard media could have an impact on T cell responses generally observed in culture. To investigate this, we tested a glucose gradient within the physiological range assaying primary T cell responses at 10 mM, a glucose concentration mimicking hyperglycaemia, but also close to the 11.1 mM concentration found in traditional cell culture media such as RPMI, at 5 mM the physiological steady state concentration found in the human circulation and at 2.5 mM associated with hypoglycaemia. In line with previous reports T cell viability in culture was not affected by changing the concentrations of glucose [29,30], even after a prolonged duration of culture (Figure S1). We found that glucose concentrations affected the frequency of cells in individual populations in the three stimulations investigated (Figure 1E). We thus set out to assess individual functional and phenotypic features in more detail.

### 2.2. Glucose Availability Differentially Affects the Effector Function of CD3-Activated versus Virus-Specific T Cells

**Figure 2:**
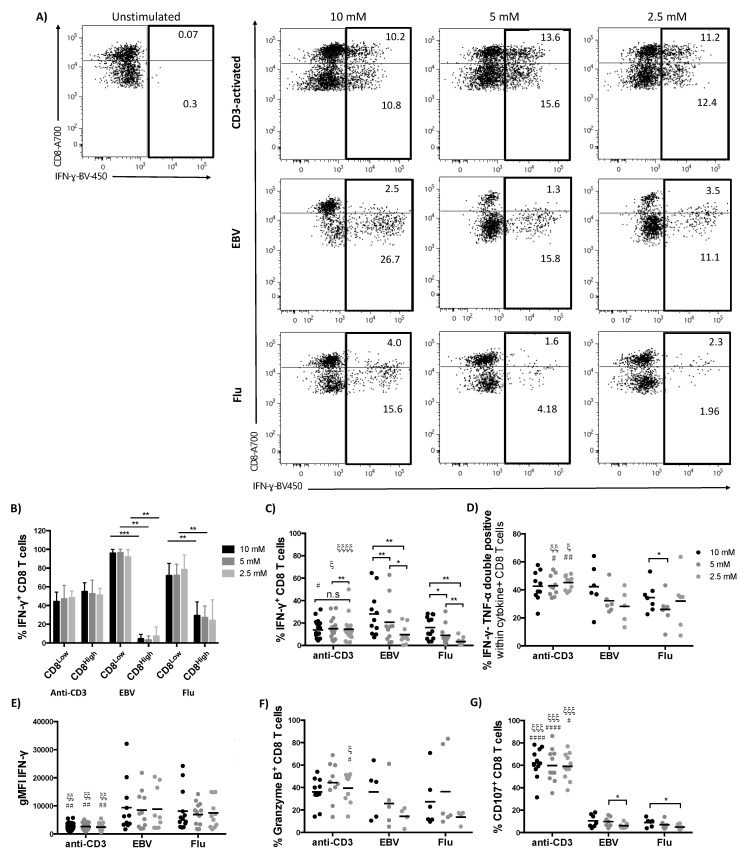
Glucose availability alters cytokine production by virus-specific but not CD3-activated CD8 T cells. T cells were stimulated as detailed in Figure 1A and cultured in the indicated glucose concentrations for the entire period of the experiment. Following overnight restimulation on day 7 cytokine production was determined. (**A**) Representative dot plots showing production of IFN-γ by CD3-activated or virus-specific CD8 T cells stimulated in different concentrations of glucose. (**B**) Distribution of IFN-γ^+^ cells into CD8^low^ and CD8^high^. (**C**) Percentage of CD8^+^ T cells producing IFN-γ and (**D**) percentage of CD8 T cells coproducing IFN-γ and TNF-α after respective stimulation. (**E**) IFN-γ geometrical mean fluorescence intensity (gMFI) in IFN-γ^+^ CD8^+^ T cells. (**F**) Percentage of granzyme B^+^ CD8 T cells and (**G**) percentage of CD107^+^ CD8 T cells after stimulation with anti-CD3, EBV and flu peptides in different concentrations of glucose. Mann–Whitney test was used when comparing between the same concentration of glucose in different groups, ξ *p* < 0.05, ξξ *p* < 0.01, ξξξ *p* < 0.001 when comparing with flu-stimulated group and # *p* < 0.05 ## *p* < 0.01 #### *p* < 0.0001 when comparing with EBV-stimulated group. Wilcoxon paired *t* test when comparing between glucose concentrations within the same group, * *p* < 0.05, ** *p* < 0.01, *** *p* < 0.001.

As previously described for human CD8 T cells ex vivo, we observed all groups separating into CD8 high and low expressing T cell populations in culture, irrespective of stimulation (Figure 2A, gating strategy in Figure S2A) [10,12,31]. Interestingly, in IFN-γ^+^ CD3-activated T cells, the proportion of cells producing IFN-γ is similar in CD8 high and low T cell populations; in contrast, in EBV-specific T cells the cytokine producing cells detected are almost exclusively CD8 low, while in Flu-specific CD8 both populations contribute, but with the CD8 low being the dominant (Figure 2A,B and Figure S2B,C). We then assessed the effect of varying the glucose concentration on cytokine production and found that the frequency of IFN-γ^+^ virus-specific CD8 T cells significantly decreased at 5 mM and 2.5 mM, while the frequency of CD3-activated IFN-γ-producing CD8 T cells slightly increased at 5 mM and decreased again at 2.5 mM, but showed no significant difference between 10 and 2.5 mM glucose concentration (Figure 2C). In line with previous studies [14] more extreme reduction of glucose levels, below 1 mM or inhibition of glycolysis by 2-deoxy-d-glucose (2DG) [11,12], significantly decreased the frequency of IFN-γ^+^ CD8 T cells in CD3-activated cells (Figure S3). To further test T cell functionality at reduced glucose concentrations, we next assessed the effect of reducing glucose availability on the simultaneous production of IFN-γ and TNF-α. Coproduction of these cytokines showed a trend to decrease in virus-specific CD8 T cells but was unaltered in CD3-activated cells (Figure 2D).

These findings further indicate that stimulation by virus-specific peptide and CD3 activation induces effector functions by different cell populations. However, at the point of restimulation, IFN-γ^+^ virus-specific and CD3 activated T cell populations are comparable in their composition of naïve, T central memory (T_CM_), T effector memory (T_EM_) and terminally differentiated effector memory RA-re-expressing (T_EMRA_) cells as defined by CCR7/CD45RA staining (Figure S4A–E), suggesting that these markers do not provide sufficient resolution to explain the observed differences [32]. The only significant change we observed was that at a glucose concentration of 2.5 mM the frequency of virus-specific T_EM_ decreased, likely contributing to the reduction in IFN-γ^+^ virus-specific cells at this concentration (Figure S4B).

Of note IFN-γ production on a per cell basis, as measured by geometrical mean fluorescence intensity (gMFI), was unaffected by glucose availability over a physiological range in all stimulation groups (Figure 2E). Generally, IFN-γ gMFI was significantly higher in EBV and flu-specific T cells compared to CD3-activated CD8 T cells irrespective of glucose concentration. The relative resistance/tolerance to variations in glucose concentration displayed by CD3-activated T cells versus virus-specific T cells could be due to the strength of TCR signalling. To manipulate signal strength, we increased/decreased the concentration of immobilized anti-CD3. Indeed, the overall amount of IFN-γ produced by CD8 T cells increased with increasing concentration of anti-CD3, however, increasing or decreasing the concentration of immobilized anti-CD3 did not alter sensitivity to glucose availability at 5 mM or 2.5 mM (Figure S5A) nor did the addition of using CD28 and CD3 for activation (Figure S4B). We found similar results when assessing anti-CD3 or anti-CD3/CD28 stimulated CD4 T cells from the same donors (Figure S5C,D).

To further investigate T cell functionality, we assessed cytotoxic capacity of IFN-γ^+^ CD8 T cells. Cytotoxic effector molecules, such as granzyme B [33] are secreted in lytic granules, which are surrounded by a lipid bilayer containing lysosomal-associated membrane glycoprotein (LAMP-1, CD107a) [34]. We found the frequency of granzyme B to be similar in virus-specific and CD3-activated T cells at 10 mM, however, it tended to decrease in virus-specific T cells at lower concentrations of glucose (Figure 2F). Strikingly, the percentage of CD107a^+^ CD8 T cells was significantly lower in CD8 T cells stimulated with viral peptides compared to CD3-activated T cells in all glucose concentrations assayed. In virus-specific T cells the proportion of CD107a^+^ CD8 T cells further decreased when glucose concentration was reduced (Figure 2G). These results could suggest impaired cytotoxic degranulation in virus-specific CD8 T cells.

### 2.3. Glucose Concentration Impacts Proliferation of CD8 T Cells

**Figure 3:**
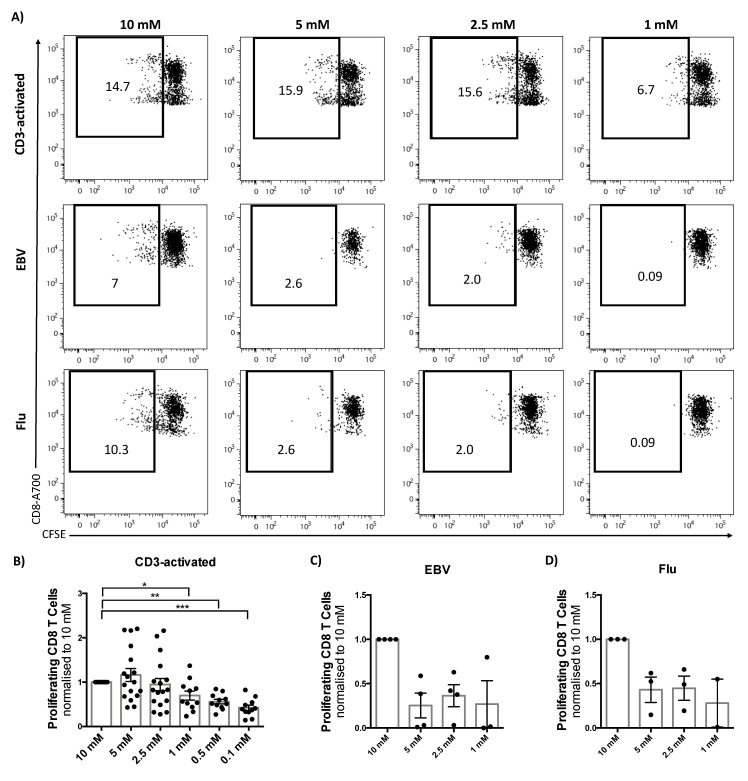
Glucose availability affects CD8 T cells proliferation. T cells were labelled with the fluorescent dye carboxyfluorescein succinimidyl ester (CFSE) to assess proliferation. Cells were stimulated for 72 h in different glucose concentrations as indicated and unstimulated cells were used as a control. (**A**) Dot plot showing CFSE dilution/T cell proliferation in different concentrations of glucose. (**B**) Fold change in proliferation of CD8 T cells normalized to proliferation in 10 mM glucose; CD3-activated, (**C**) EBV- and (**D**) flu-stimulated T cells in different concentrations of glucose. Mean ± SEM. Wilcoxon paired *t* test, * *p* < 0.05, ** *p* < 0.01, *** *p* < 0.001.

The observation that the frequency of IFN-γ producing cells, but not the amount of cytokine produced per cell, was affected by glucose concentration indicated that cell proliferation might be an important contributor to the observed changes. Indeed, in line with the reduced frequency of IFN-γ^+^ T cells, reducing glucose from 10 to 5 mM was enough to decrease proliferation in virus-specific T cells, while in CD3-activated cells proliferation was only affected when glucose concentration was lower than 1 mM (Figure 3A–D). These results suggest that although virus-specific T cells are able to produce IFN-γ at higher levels than CD3-activated cells their proliferation capacity in lower glucose concentrations is impaired, impacting on the frequency of IFN-γ^+^ CD8 T cells. 

### 2.4. The Activation Phenotype of CD3-Activated and Virus-Specific T Cells is Differentially Impacted by Glucose Availability

**Figure 4:**
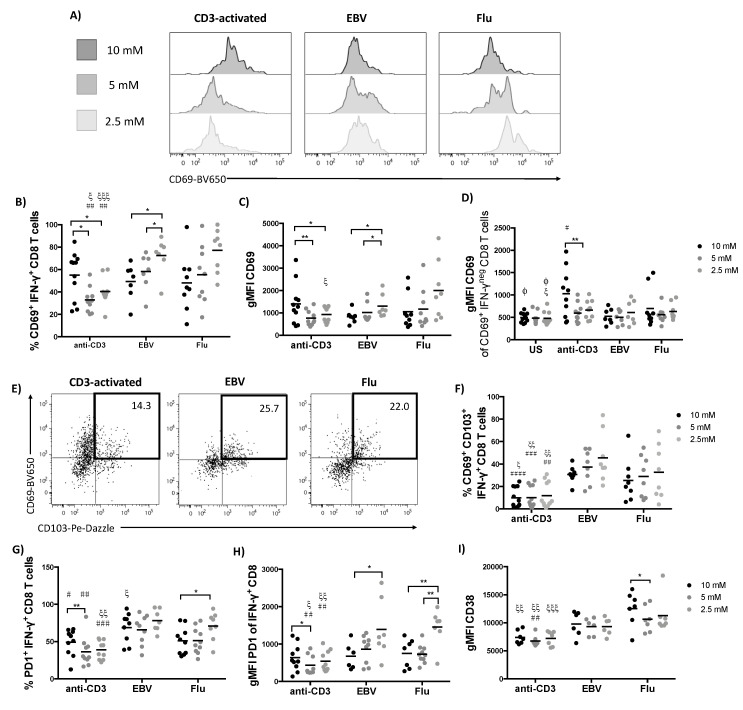
Glucose restriction induces markers of residency in virus-specific T cells. Activation-related markers were assessed in IFN-γ^+^ CD8 T cells on day 7 post restimulation. (**A**) Representative histogram of CD69 expression in IFN-γ^+^ CD8 T cells. (**B**) Percentage and (**C**) gMFI of CD69 in IFN-γ^+^ and (**D**) in IFN-γ^neg^ CD8 T cells. (**E**) Representative dot plot showing CD103 and CD69 expression in IFN-γ^+^ CD8 T cells after stimulation in media containing 10 mM of glucose. (**F**) Percentage of CD69^+^ CD103^+^ double positive IFN-γ^+^ CD8 T cells. (**G**) Percentage and **(H**) gMFI of coinhibitory PD1. (**I**) Expression level of the activation marker CD38. Mann–Whitney test when comparing between the same concentration of glucose in different groups, Φ *p* < 0.05 when comparing with CD3-stimulated, ξ *p* < 0.05, ξξ *p* < 0.01, ξξξ *p* < 0.001 when comparing with flu-stimulated group and # *p* < 0.05, ## *p* < 0.01, ### *p* < 0.001, #### *p* < 0.0001 when comparing with EBV-stimulated group. Wilcoxon paired *t* test when comparing between glucose concentrations within the same group, * *p* < 0.05, ** *p* < 0.01.

Since we observed differences in CD8 expression and cytokine production we sought to further interrogate the phenotype observed in our tSNE analysis by comparing the phenotype of CD3-activated and virus-specific T cells. First, we investigated the expression of the early activation marker CD69. CD69, by virtue of its ability to suppress lymphocyte egress via inhibition of sphingosine 1-phosphate sensing is associated with tissue resident memory (T_RM_) T cells in multiple tissues [10,12,31]. Despite their reactivation in vitro and increased production of cytokine, IFN-γ^+^ virus-specific CD8 T cells expressed similar levels of CD69 to their CD3-activated counterparts at hyperglycaemic glucose concentrations (10 mM; Figure 4A,B). However, while lowering glucose levels led to a reduced frequency of CD69^+^ CD3-activated T cells, the opposite was true for EBV-specific T cells with both frequency and gMFI significantly increasing (Figure 4A–C). Flu-specific T cells also showed a trend for CD69 levels to increase with decreasing glucose concentration at low 2.5 mM. While changes in glucose concentration had no effect on the expression of CD69 in CD8 T cells left unstimulated or on the IFN-γ negative T cells from the peptide stimulated culture wells, CD69 was increased IFN-γ^neg^ population in wells coated with immobilized anti-CD3 (Figure 4D). This might be attributed to cytokines produced by responding cells or by the fact that all cells in these wells would have been in contact with the coating antibody. The increased CD69^+^ expression in virus-specific CD8 T cells in lower concentrations of glucose could point to an adaptation allowing cells to remain at a site of inflammation where glucose is locally depleted [14]. We thus also probed for coexpression of another prototypical residency marker, CD103, which is implicated in tissue retention via its capacity to tether memory T cells to epithelial E-cadherin [11,12]. The frequency of CD69^+^ CD103^+^ double positive CD8 T cells was significantly higher in virus-specific T cells compared to CD3-activated T cells with a trend for this double positive population to further increase in lower glucose concentrations (Figure 4E–F), supporting the idea of virus-specific CD8 T cells adopting a T_RM_ phenotype. TCR-stimulation was key to inducing increased frequencies of CD103 expressing cells, as changes to glucose concentration in the absence of stimulation had no effect (data not shown).

Another important activation marker and hallmark coinhibitory receptor of exhausted T cells is PD-1. PD-1 expression showed a similar pattern to CD69, showing decreased frequency and expression level on CD3-activated T cells and upregulation in virus-specific T cells in response to decreasing glucose concentration (Figure 4G–H). Incidentally, PD-1 has been reported to be highly expressed on T_RM_, despite these cells being fully functional [10] in line with our finding that EBV-specific CD8 T cells express increased levels of both PD-1 and IFN-γ^+^ compared to CD3 activated cells. Reduced glucose conditions specifically induced upregulation of PD-1 and CD69 but did not increase expression of other activation markers such as CD38 (Figure 4I) and HLA-DR (Figure S6).

### 2.5. The Expression of Nutrient Transporters is Influenced by Glucose Concentration and is Significantly Higher in Virus-Specific Compared to CD3-Activated T Cells 

**Figure 5:**
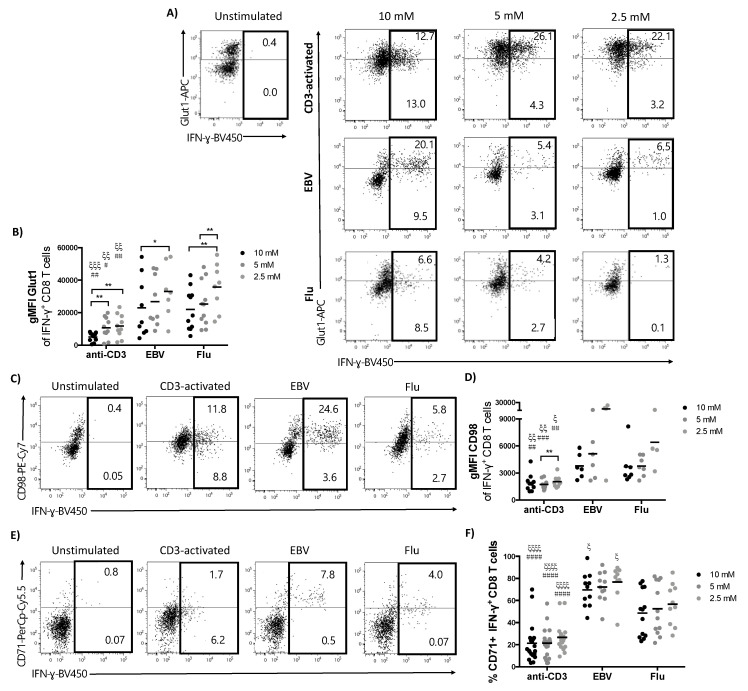
Virus-specific T cells have a highly activated metabolic profile compared to CD3-activated T cells. T cells were stimulated as outlined in Figure 1A. Metabolic markers were assessed upon restimulation on day 7 in the responding IFN-γ^+^ CD8 T cell population. (**A**) Representative dot plots showing Glut1 expression and IFN-γ production by CD8 T cells after stimulation in media containing different concentrations of glucose. (**B**) gMFI of Glut1 in the IFN-γ^+^ CD8 T cell population. (**C**) Representative dot plots showing CD98 expression and IFN-γ production by CD3-activated or virus-specific CD8 T cells after stimulation in media containing 10 mM of glucose and (**D**) summary data showing gMFI of CD98 in IFN-γ^+^ CD8 T cells at all glucose concentrations. (**E**) Representative dot plot of CD71 expression in 10 mM glucose and (**F**) percentage of CD71^+^ IFN-γ^+^ CD8 T cells in different concentrations of glucose. Mann–Whitney test when comparing between the same concentration of glucose in different groups, ξ *p* < 0.05, ξξ *p* < 0.01, ξξξ *p* < 0.001, ξξξξ *p* < 0.0001 when comparing with flu-stimulated group and # *p* < 0.05 ## *p* < 0.01 ### *p* < 0.001, #### *p* < 0.0001 when comparing with EBV-stimulated group. Wilcoxon paired *t* test when comparing between glucose concentrations within the same group, * *p* < 0.05, ** *p* < 0.01, *** *p* < 0.001.

Next we investigated the impact of reducing glucose on the expression profile of key nutrient transporters in our three differentially stimulated CD8 T cell populations, since we reasoned that decreased availability of glucose might induce increased expression as a compensatory mechanism. We focused on three transporters, one of the major glucose transporters functional in CD8 T cells, Glut1, the neutral amino acid transporter CD98 (Slc7A5) and CD71 (transferrin receptor 1) responsible for allowing cells to regulate iron uptake. All three transporters have been shown to be vital for T cell function [6,14,35,36]. We found that Glut1 expression was significantly higher in virus-specific compared to CD3-activated T cells at all glucose concentrations applied (Figure 5A,B). 

Glut1 expression per cell increased with decreasing concentration of glucose in all populations, likely indicating that T cells were maximizing glucose uptake under limiting conditions (Figure 5A,B). A decrease of glucose concentrations also impacted on amino acid uptake, leading to a corresponding increase in expression of CD98 (Figure 5C,D), which has been shown to indicate increased uptake of amino acids such as alanine by CD8 T cells [32]. Again, CD98 was expressed at a significantly higher level in virus-specific compared to generically activated T cells (Figure 5D). Finally, we found expression of CD71 to be the most distinct between the populations. The frequency of CD71 positive cells was low in CD3 activated, intermediate in Flu-specific and high in EBV-specific cells (Figure 5E,F) demonstrating further phenotypic differences not only between CD3 activated versus virus-specific, but also between virus-specific T cells targeting different pathogens. These results are intriguing as although virus-specific T cells express higher levels of nutrient transporters compared to CD3-activated cells, this did not confer increased resistance to changes in glucose concentration, as evidenced by the significant negative impact on frequency of IFN-γ positive and proliferation of virus-specific CD8 in response to peptide stimulation (Figure 3). 

### 2.6. T Cells Cultured in Glucose Mimicking Physiological Concentrations are More Susceptible to Viral Infection

**Figure 6:**
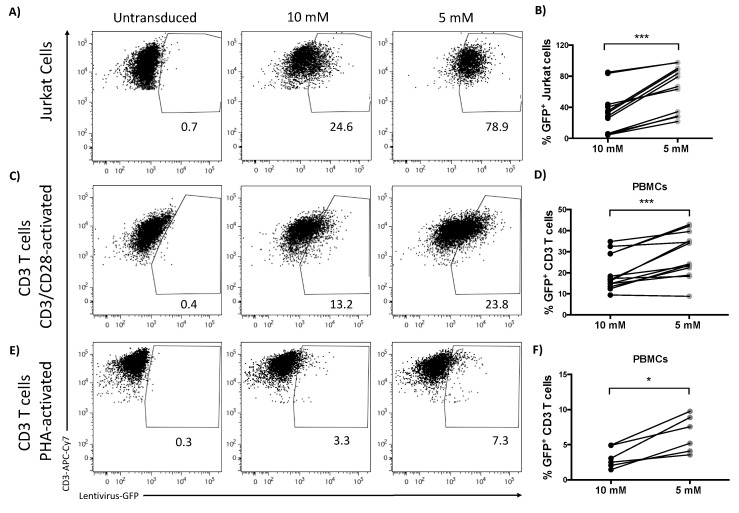
Lowering glucose levels increases the susceptibility of T cells to lentiviral transduction. Jurkat cells and PBMCs were transduced and maintained in 10 or 5 mM of glucose with a lentivirus containing GFP protein, expression of which was detected to follow transduction efficiency. (**A**) Representative dot plot of untransduced and transduced Jurkat cells in different concentrations of glucose. (**B**) Percentage of GFP^+^ Jukat cells after lentiviral transduction in different concentrations of glucose. (**C**) PBMC were preactivated for 48 h and GFP^+^ CD3 T cells were detected 3 days after lentiviral transduction, representative dot plot, (**D**) and percentage of GFP^+^ CD3 T cells after lentiviral transduction in the glucose concentrations indicated. (**E**) Representative dot plot of transduced CD3 T cells after activation with PHA for 72 h and (**F**) percentage of GFP^+^ CD3 T cells transduced after PHA stimulation Wilcoxon paired *t* test, * *p* < 0.05, *** *p* < 0.001.

It has been described that T cell tropic viruses preferentially target metabolically active cells as a strategy to maximize viral propagation [15,16]. Glut1 is being utilized as an entry receptor by the human T lymphotropic virus (HTLV) [37,38] and Glut1 expression was also increased on T cells harbouring HIV [39]. We thus wondered whether culturing T cells in conditions mimicking physiological glucose concentrations would lead to increased viral susceptibility, since viral evolution likely favoured infection conditions present in vivo. To address this, we examined the potential to transduce T cells using lentiviral vectors in vitro under physiological glucose concentrations not normally seen in traditional culture. First, we tested our hypothesis that T cell infectivity would be enhanced at 5 mM glucose in the Jurkat cell line, which is more readily transduced with lentiviral vectors than primary cells. Reducing the glucose concentration from 10 to 5 mM significantly increased transduction efficiency of Jurkat cells (Figure 6A,B). Next we repeated the experiment to assess the effect of glucose availability on the efficiency to transduce primary human T cells. Indeed, transduction of primary T cells was significantly enhanced at 5 mM glucose concentration compared to 10 mM typically seen in culture media (Figure 6C,D). To investigate whether this finding was influenced by the type of stimulation used for activation we alternatively stimulated T cells with phytohaemagglutinin (PHA). Again we found an increase in transduction efficiency when glucose levels were reduced (Figure 6E,F). In line with the finding for increased levels of Glut1 in HIV infection [39,40], transduced T cells showed increased levels of Glut1 compared to non-infected T cells (Figure S7).

## 3. Discussion

Due to the central role that cellular metabolism plays in enabling immune responses, it is pertinent to assess how local conditions impact the T cell response. Nutrient availability influences T cell function, impacting on the immune response at the site of inflammation, infection and in the tumour microenvironment [14]. Previous studies have shown that composition of the culture media can profoundly affect experimental results [16,41]. Here we explored the impact of adjusting the glucose concentration, to mimic physiological levels, on primary human T cells in culture. Even seemingly modest alterations to glucose concentration significantly altered T cell phenotype and function. Importantly, we found a differential effect on virus-specific responses, activated via peptide stimulation, versus the basic generic activation by immobilized CD3. Limiting glucose to physiological levels significantly reduced the frequency of IFN-γ producing virus-specific but not CD3-activated T cells. In both groups, cytokine production on a per cell basis remained unchanged. However, more extreme glucose depletion to below 1 mM affected proliferation of CD3-activated T cells, resulting in a reduced frequency of cytokine producing cells (Figure S3).

T cell stimulation in physiological glucose conditions led to increased expression of key nutrient transporters. Mirroring the previous observation that depletion of amino acids led to enhanced expression of the amino acid transporter CD98 [32], reduction in glucose availability resulted in a general trend for increased expression of Glut1. In a recent study Klein-Geltink et al. demonstrated that T cells originally primed in the presence of 11 mM glucose prior to short-term glucose restriction (1 mM glucose for 20 h were functionally superior when transferred back into 10 mM hyperglycaemic culture conditions compared to T cells not exposed to a period of glucose deprivation. Interestingly, T cells experiencing a period of glucose restriction exhibited sustained metabolic changes, with an enhanced capacity to use OXPHOS and high expression of Glut1 [37], suggesting sustained functional changes induced by nutrient availability.

In our experiments, the virus-specific T cells would have been naturally primed and differentiated in vivo, before being reactivated in vitro, thus any “metabolically imprinted” changes are likely already present preculture. This would also apply to the mixture of polyclonal T cells (re)activated by CD3 and indeed is the case for most human studies and therapeutic applications.

Intriguingly, we found that the glucose transporter Glut1 was especially highly expressed on T cells specific to chronic EBV, independent of the glucose concentration in the media. Previous studies have shown T cells expressing increased Glut1 to have an enhanced capacity to release IFN-γ [15], indeed EBV-specific T cells were able to produce large quantities of IFN-γ, as measured by IFN-γ gMFI on a per cell basis. Counterintuitively, the high expression of Glut1 did not lead to better resistance to limiting levels of glucose, as EBV-specific T cells were highly sensitive to glucose restriction and high Glut1, CD98 and CD71 expression could not rescue this phenotype (Figure 4). We found a similar trend for Flu-specific T cells, although this was not as pronounced. In contrast, polyclonal T cells preferentially activated by CD3 in culture constitute a population more flexible in their metabolic requirements and defects in function only become apparent at supraphysiological glucose concentrations, below 1 mM. Since CD3 activation is used during the generation of therapeutic T cells it will be interesting to understand the determinants of this metabolic plasticity. 

Of note, despite their distinct responses, virus-specific and CD3-activated IFN-γ producing T cells were composed of similar frequencies in terms of naïve, memory, effector and EMRA T cells (Figure S4). Interestingly, limiting glucose availability exclusively impacted on the frequency of peptide stimulated virus-specific T effector cells (Figure S4). 

We have previously described metabolic alterations in T cells specific for chronic Hepatitis B Virus (HBV), characterised by high expression of Glut1, however, this did not correlate with a superior function, but on the contrary in the case of HBV-specific T cells appeared to be a feature of exhaustion and correlated with increased expression of PD-1 and decreased propensity to produce IFN-γ [37]. In agreement with our findings, Vardhana et al. describe that repetitive stimulation of T cells leads to increased Glut1 expression and functional exhaustion [8]. Repetitive stimulation could contribute to the distinctive features of EBV-specific T cells inducing the heightened Glut1, CD98 and CD71 expression. Intriguingly, EBV and the related Karposi’s Sarcoma-Associated Herpesvirus (KSHV) can induce increased glycolysis in their host cells, possibly locally depriving T cells of glucose during viral reactivation [16]. However, as opposed to the situation in chronic HBV, effector functions in EBV-specific T cells might be preserved due to the absence of stimulation by viral antigen during phases of EBV latency [38]. In contrast, influenza virus is usually cleared, and flu-specific T cells have a less pronounced phenotype, somewhere in between CD3-activated and EBV-specific cells.

Another surprising observation we made was the impact of glucose availability on expression of CD69. CD3 activated and virus-specific T cells showed exactly opposite responses. The frequency of CD69^+^ cells within the CD3-activated T cell population significantly decreased in low glucose, while in EBV-specific T cells both the frequency and expression level of CD69 significantly increased. Coexpression of a further prototypical resident memory T cell marker, CD103 was significantly higher on virus-specific T cells compared to CD3-activated T cells and showed a trend to further increase upon glucose restriction. We speculated that lower glucose levels, for instance at the site of infection, are able to induce a reprogramming event within virus-specific T cells promoting tissue retention. Taken together, it is plausible that glucose restriction contributes to the development of a resident memory phenotype and therefore promotes retention of antigen-specific T cells at the site of an infection or tumour.

The data presented here show that virus-specific T cells are exquisitely sensitive to glucose availability despite being highly metabolically active.

If these findings translate to the in vivo situation, they would suggest that circulating virus-specific CD8 T cells are capable of producing copious amounts of effector cytokines while their proliferation is likely kept in check. Additionally, they might be more amenable to regulation through increased expression of coinhibitory PD-1.

In contrast to the alterations observed in virus-specific T cells, our data indicate that lowering glucose availability for the mixed T cell population activated by immobilized CD3 in vitro drives enhanced metabolic activation while limiting the frequency of coinhibitory PD-1 expressing T cells within the effector cytokine producing population. This could be exploited when using polyclonal T cell populations as starting material for therapeutic T cell production. Chimeric antigen receptor (CAR) modified and T cell receptor engineered T cells are commonly activated and expanded via CD3, thus culturing these in physiological glucose concentrations could reduce expression of activation markers such as immune suppressive PD-1, while neither compromising effector cytokine production nor proliferation. In addition, the upregulation of key nutrient transporters will likely increase the metabolic competitiveness of the therapeutic T cells possibly making them more resistant to the challenging tumour microenvironment.

Finally, we considered a scenario where T cells become viral hosts. Viruses targeting T cells include the human pathogens HIV and HTLV. Interestingly, the entry-receptor for HTLV-1 is Glut1 [38], while HIV preferentially infects metabolically active T cells, and increased Glut1 expression is characteristic for HIV-infected cells [15]. In line with these viral preferences, we found that T cells cultured in physiological glucose concentrations were more efficiently transduced by the lentiviral vector, than those cultured in conventional high glucose concentrations. Although different mechanisms might underlie lentiviral T cell transduction when compared to T cell infections with pathological viruses, our results did raise the possibility that studies looking at T cells in their role as viral hosts could benefit from adjusting glucose concentrations to mimic more closely the in vivo milieu. Additionally, transduction of small T cell populations, for example those specific for a given virus or tumour antigen, could potentially be enhanced by this simple change to the culture media. 

Going forward it will be important to keep in mind the T cell starting population and stimulus used for experimentation, as we show here that the differentiation status acquired in vivo significantly influenced the response to nutrient deprivation. However, the exact mechanism controlling the divergent responses of CD3 activated and virus-specific T cells remains to be determined.

## 4. Materials and Methods 

### 4.1. Healthy Donors

Blood samples from healthy volunteers were obtained under The Guy’s and St Thomas’ License (license number 12121). Each participant gave written informed consent. All storage of samples obtained complied with the requirement of the Data Protection Act 1998 and the Human Tissue Act 2004, issued by the UK parliament. All subjects gave their informed consent for inclusion before they participated in the study. The study was conducted in accordance with the Declaration of Helsinki, and the protocol for healthy volunteer recruitment and sampling was approved by the committee of the Infectious Diseases Biobank of King’s College London with reference number AS1-280119. The approval was granted under the terms of the Infectious Disease Biobank’s ethics permission (REC reference 19/SC/0232) granted by the South-Central Hampshire B Research Ethics Committee. The influence of gender and age of human subjects that took part in this study was not considered (Table 1). The gender and age of donors whose blood was obtained from cones was not collected.

### 4.2. Cell Culture

Peripheral blood mononuclear cells (PBMCs) were isolated from heparinized whole blood using density gradient centrifugation (Lymphoprep, STEMCELL Technologies, Cambridge, UK). Cells were either used for directly or cryopreserved in liquid nitrogen in foetal calf serum (FCS) with 10% dimethyl sulfoxide (DMSO; Sigma-Aldrich, Darmstadt, Germany) for later experimentation. In glucose free RPMI 1640 medium supplemented with 0.1 mM non-essential amino acids, 10 mM HEPES buffer, 1 mM sodium-pyruvate, 50 IU/mL of streptomycin and penicillin (all from Sigma-Aldrich, Darmstadt, Germany) and 10% FCS, 0.5 × 10^6^ PBMCs per mL were resuspended. Glucose free RPMI was reconstituted with 10, 5 or 2.5 mM of D-glucose (Sigma-Aldrich, Darmstadt, Germany). Cells were kept in an incubator at 37 °C, 5% CO_2_.

### 4.3. Stimulation of Viral Responses

Of ProMix peptide pools for Influenza (PX-FLU) and Epstein–Barr virus (PX-EBV) covering common HLA-A and HLA-B types (ProImmune, Oxford, UK) 0.5 µg/mL were added together with 20 IU/mL of interleukin (IL)-2 (Miltenyi Biotec, Bergisch Gladbach, Germany) to cells cultured in media containing 10, 5 or 2.5 mM of glucose. After 72 h half of the media was replaced with fresh media containing the corresponding concentration of glucose and IL-2 was again adjusted to 20 IU/mL final concentration. On day 7, 0.5 µg/mL of Flu or EBV peptide pool and 1 µg/mL of Brefeldin A (BFA; Biolegend, San Diego, CA, USA) were added to restimulate the cells overnight. Unstimulated cells incubated in the same culture conditions were used as control. Sixteen hours after restimulation cells were stained for flow cytometry analysis.

### 4.4. Stimulation with Plate-Bound Antihuman CD3 Antibody

Ninety-six well plates were coated with 1 µg/mL of antihuman CD3 antibody (anti-CD3) diluted in phosphate buffered saline (PBS), unless stated otherwise. After two hours, the wells were washed with PBS and the cells were added in the corresponding media containing different concentrations of glucose and 20 IU/mL of IL-2. After overnight incubation, cells were moved to non-coated wells, 72 h after the first stimulation, fresh IL-2 was added in the appropriate glucose concentrations. Cells were moved into fresh anti-CD3 coated wells on day 7 for restimulation overnight in the presence of 1 µg/mL of BFA. Where indicated, 5 mM 2-deoxy-d-glucose was added during the second overnight stimulation. Unstimulated cells were incubated in the same culture conditions were used as control. Sixteen hours after stimulation, cells were stained for flow cytometry analysis.

### 4.5. Flow Cytometry

Cells were washed with PBS and stained for surface markers with fluorochrome-labelled antibodies in PBS and live/dead dye (1.5 µL/mL, ThermoFisher Scientific, Waltham, MA, USA) for 30 min, at 4 °C and protected from light. The antibodies used for extracellular staining were: CD3 APC-Cy7, CD8-Alexa 700 (A700), CD69 Brilliant Violet (BV)-605, PD-1-PE, CD71-Per-CP-Cy7, CD38-BV-510 or CD38 PE-Dazzle, CD4 PE-Cy7, HLA-DR-BV-510, CD103 PE-Dazzle, CD45RA-BV450, CCR7-FITC (all from BioLegend, San Diego, California, USA) and CD98 PE-Cy7 (Miltenyi Biotech, Bergisch Gladbach, Germany). Cells were washed with PBS and fixed with fix/perm buffer (BD Biosciences) for 20 min at 4 °C and protected from light followed by 30 min incubation with permeabilization buffer (1% FCS, 0.1% saponin in PBS) containing intracellular antibodies, Glut1-APC (Abcam, Cambridge, UK), TNF-α-FITC, Granzyme B-FITC (BioLegend) and IFN-γ-BV-450 (Becton Dickinson, Franklin Lakes, New Jersey, USA). For CD107a-PE (BioLegend) staining, the antibody was added and monensin (1 µg/mL) at the time cells were restimulated. After staining, samples were then washed with PBS and maintained at 4 °C until they were acquired with a BD LSR Fortessa 3 (Becton Dickinson). For staining of the nuclear protein Ki-67-APC (BioLegend), FOXP3 buffer (Becton Dickinson) was used following the manufacturer’s instructions. FlowJo 8.8.6 and 10.5.3 software was used for the analysis.

### 4.6. Proliferation Assay

Carboxyfluorescein succinimidyl ester (CFSE) (ThermoFisher Scientific, Waltham, MA, USA) was reconstituted following the manufacturer’s instructions. PBMCs resuspended in PBS were incubated with 1 µM CFSE for 20 min at 37 °C. FCS was added to each well to stop the reaction and cells were incubated 5 min at 37 °C. Cells were washed extensively and fresh media were added to distribute the cells in the different conditions. Cells were stimulated with plate-bound anti-CD3, 0.5 µg/mL of Flu or EBV peptide in the presence of 20 IU/mL of IL-2 for 72 h. Proliferation was assessed by CFSE dilution using flow cytometry.

### 4.7. Production of Lentiviral Particles

Lentiviral particles were obtained by transfecting HEK293T cells with three plasmids encoding the envelope (VSC-g), packaging genes (Gag-pol) and one lentiviral plasmid containing the GFP protein and an internal promoter (pCSGW). HEK293T cells were plated in a Petri dish, 25 µM of chloroquine was added to the plates before transfection. Of CaCl_2_ (1.25 M) and H_2_O 200 μL were added to the DNA and 1 mL of 2× HBS. The solution was added dropwise to the plates containing the HEK293T cells. The plates were swirled gently and kept in the incubator for 5 h. Cells were then washed with phosphate buffered saline (PBS) and 10 mL of fresh DMEM media was added to each plate. After 48 h in the incubator, the supernatant was filtered through a 45 μM filter and frozen at −80 °C in 1 mL aliquots. The lentiviral vector was titrated using Jurkat cells.

### 4.8. Lentiviral Transduction of Jurkat Cells

Jurkat cells were transduced with the obtained lentiviral particles using 1 µg/mL of polybrene in media containing different concentrations of glucose, 10 mM, 5 mM or 2.5 mM. Lentiviral soup was added to the cells in a 1:1 ratio. Twenty-four hours after, cells were washed to remove the lentiviral particles and 24 h after the samples were acquired in the flow cytometer to detect expression of GFP and Glut-1 transporter after staining with Glut-1-APC antibody as explained before.

### 4.9. Lentiviral Transduction of CD3/CD28-Activated PBMCs

PBMCs were stimulated 48 h with 1 µg/mL of plate-bound anti-CD3/antiCD28 in the presence of 100 IU/mL of IL2. After 48 h, cells were transferred to uncoated wells and were transduced with lentivirus containing GFP by mixing in the presence of 1 µg/mL polybrene in media containing either 10 or 5 mM of glucose. Lentiviral soup was added to the cells in a 1:1 ratio. Seventy-two hours after, the GFP signal was detected by flow cytometry.

### 4.10. Lentiviral Transduction of PHA-Activated PBMCs

PBMCs were stimulated for 72 h with 5 µg/mL of phytohaemagglutinin A (PHA) in the presence of 50 IU/mL of IL2. After 72 h, cells were washed and transduced with a lentivirus as explained before. 72 h after, GFP signal was detected in the transduced cells by flow cytometry.

### 4.11. Statistical Analysis

Prism software was used for the statistical analysis. A paired *t*-test and non-parametric Wilcoxon matched-pairs signed rank test were performed when comparing different concentrations of glucose within the same stimulus. An unpaired *t*-test and non-parametric Mann–Whitney test were used when comparing results from the same concentration of glucose between different kinds of stimulation, as indicated in the figure legends.

## Figures and Tables

**Table 1:** Demographics of the healthy donors recruited for the study.

Gender	Number	Age
Male	11	36 (22–59)
Female	10	38 (22–58)
Unknown	7	N/A

## Supplementary Materials

The following are available online at https://www.mdpi.com/2218-1989/10/11/461/s1, Figure S1: Reduced glucose availability does not affect T cell viability, Figure S2: Gating strategy to detect IFN-γ^+^ cells after different stimulus, Figure S3: IFN-γ production by CD8 T cells is reduced upon glucose restriction after stimulation with immobilized anti-CD3, Figure S4: Glucose availability differentially affects differentiation of CD-3 activated and viral specific T cells, Figure S5: The strength of TCR stimulation with immobilized CD3 does not affect IFN-γ production by T cells in different glucose concentration, Figure S6: Glucose concentration does not affect HLA-DR expression in activated CD8 T cells, Figure S7: Glut1 levels are increased in transduced primary T cells.
