# Supplementary material for: Divergent Impact of Glucose Availability on Human Virus-Specific and Generically Activated CD8 T Cells
Source: Metabolites. 2020 Nov 13;10(11):461. doi: 10.3390/metabo10110461 (PMC7696163; doi:10.3390/metabo10110461)
Supplement: Supplementary file 1 [file metabolites-10-00461-s001.pdf]

metabolites

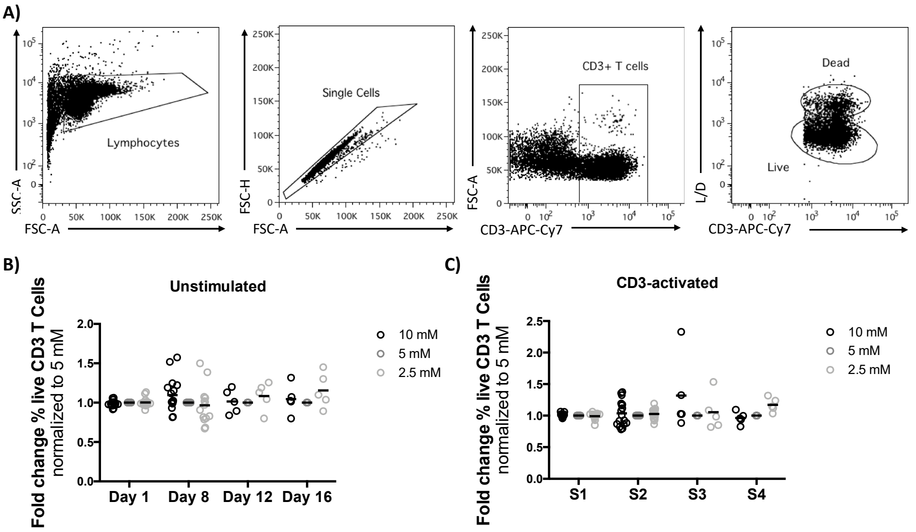

Figure S1. Reduced glucose availability does not affect T cell viability. T cells were maintained with or without stimulation in presence of IL-2 for 16 days in media containing 10, 5 or 2.5 mM of glucose. **A)** Representative gating strategy followed to identify live and dead CD3 T cells at different time points. **B)** Fold change of the percentage of live CD3 T cells normalized to the percentage of live cells detected at 5 mM in unstimulated and **C)** anti-CD3 stimulated CD3 T cells. Wilcoxon paired t test.

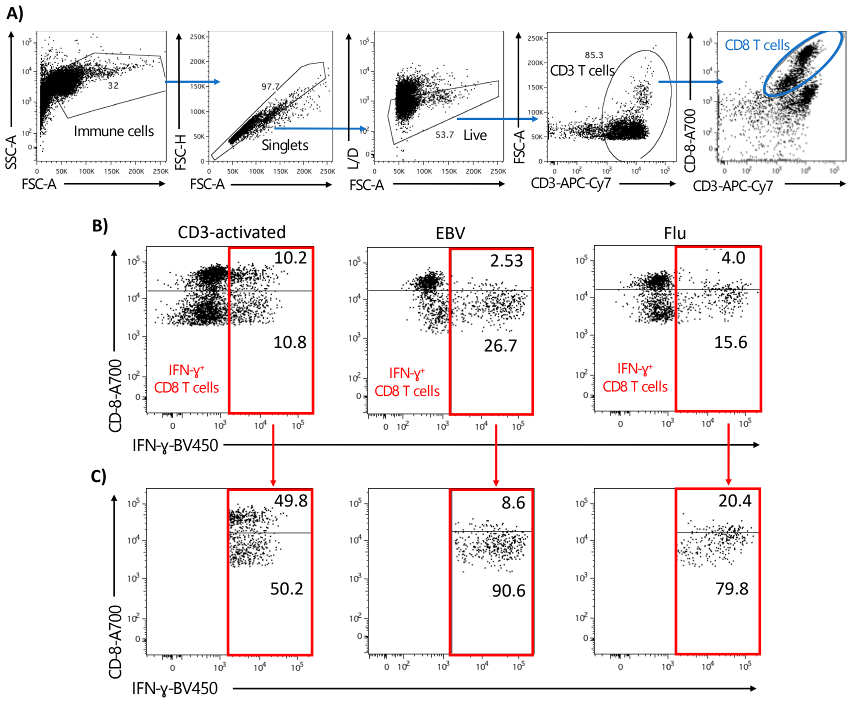

Figure S2. Gating strategy to detect IFN-γ^{+} cells after different stimulus. A) Within the immune cells, singlets were gated using FSC-A vs FSC-H. Live/dead dye was used for dead cell exclusion, allowing the detection of live cells. CD3 T cells, followed by CD8 T cells, were detected in the live populations. **B)** As shown in all the figures in the manuscript, IFN-γ^{+} CD8 T cells were detected in CD3-stimulated, EBV and Flu-specific CD8 T cells. **C)** Dot plots showing the percentage of IFN-γ^{+} CD8 high and low, within the IFN-γ^{+} CD8 T cells. The percentage of the two populations of IFN-γ^{+}

cells expressing different levels of CD8 was investigated within the IFN-γ⁺ cells (Figure 2B), as all the summary data shown throughout the manuscript.

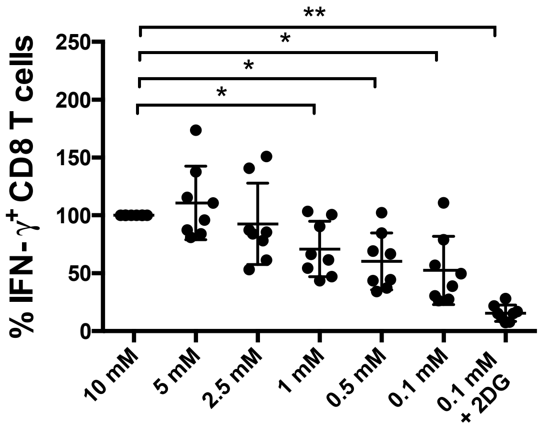

Figure S3. IFN-γ production by CD8 T cells is reduced upon glucose restriction after stimulation with immobilized anti-CD3. Percentage of IFN-γ⁺ CD8 T cells in CD3-activated cells in presence of different concentrations of glucose. Wilcoxon paired t test, *p<0.05.

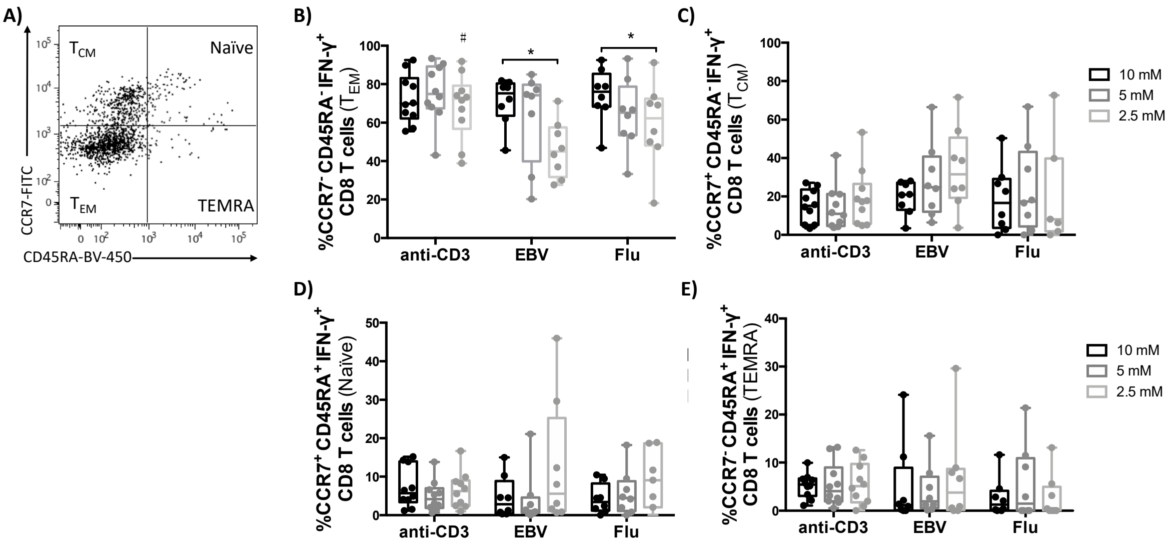

Figure S4. Glucose availability differentially affects differentiation of CD-3 activated and viral specific T cells. A) CD45RA and CCR7 were used to define the **B)** effector memory (T_{EM}) **C)** central memory (T_{CM}), **D)** naïve and **E)** terminally differentiated effector memory RA-re-expressing (TEMRA) CD8 T cells after the indicated stimulation in media containing different concentrations of glucose.

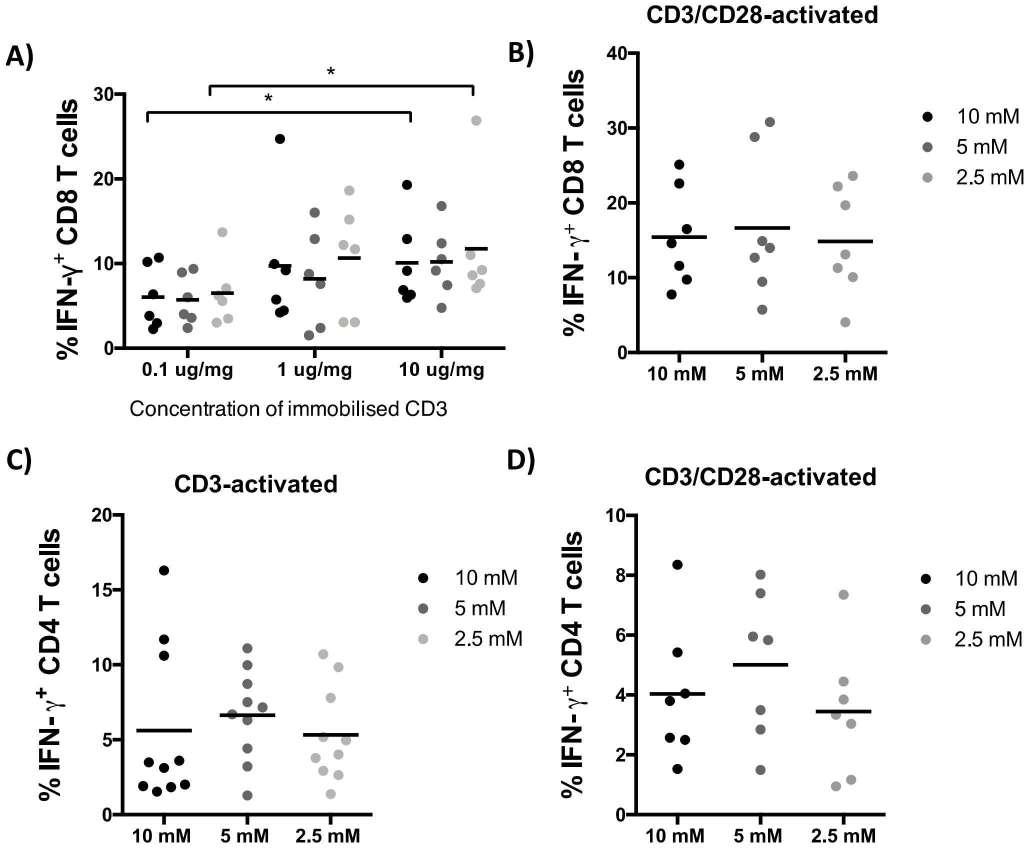

Figure S5. The strength of TCR stimulation with immobilized CD3 does not affect IFN-γ production by T cells in different glucose concentration. A) Percentage of IFN-γ CD8 T cells after stimulation with different concentrations of immobilized anti-CD3 in presence of different concentrations of glucose. **B)** Percentage of IFN-γ CD8 T cells after stimulation with 1 μg/ml of immobilized anti-CD3 and 1 μg/ml CD28. **C)** Percentage of IFN-γ in CD3-activated and **D)** CD3/CD28-activated CD4 T cells. Wilcoxon paired t test, *$p<0.05$.

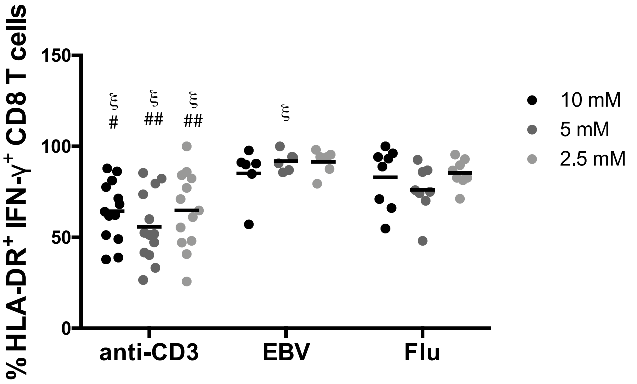

Figure S6. Glucose concentration does not affect HLA-DR expression in activated CD8 T cells. Percentage of HLA-DR⁺ IFN-γ⁺ CD8 T cells in CD3, EBV and flu-stimulated CD8 T cells. Mann-Whitney test when comparing between the same concentration of glucose in different groups, ξ $p<0.05$ when comparing with flu-stimulated group and # $p<0.05$ ## $p<0.01$ when comparing with EBV-stimulated group.

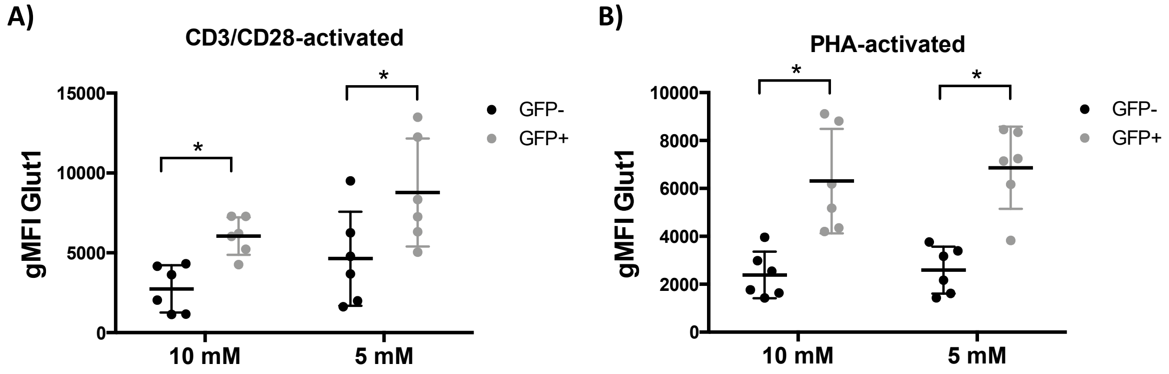

Figure S7. Glut1 levels are increased in transduced primary T cells. A) gMFI of Glut1 in non-infected and lentiviral-infected T cells in 10 and 5 mM of glucose after CD3/CD28 and **B)** PHA stimulation. Wilcoxon paired t test, $^*p<0.05$.
